# TGF-β induces liver fibrosis via miRNA-181a-mediated down regulation of augmenter of liver regeneration in hepatic stellate cells

**DOI:** 10.1371/journal.pone.0214534

**Published:** 2019-06-05

**Authors:** Parul Gupta, Teja Naveen Sata, Ajay K. Yadav, Amit Mishra, Nisha Vats, Md. Musa Hossain, M. G. Sanal, Senthil Kumar Venugopal

**Affiliations:** 1 Faculty of Life Sciences and Biotechnology, South Asian University, Akbar Bhawan, Chanakyapuri, New Delhi, India; 2 Department of Molecular and Cellular Medicine, Institute of Liver and Biliary Sciences, New Delhi, India; National Institutes of Health, UNITED STATES

## Abstract

**Objective:**

To study the role of miRNA-181a and augmenter of liver regeneration in TGF-β-induced fibrosis in hepatic stellate cells.

**Methods:**

LX2 cells were treated with 20 ng/ml TGF-β for 24 h. miRNA-181a, ALR plasmid and empty vectors were transfected using siPORT NeoFx reagent. Cells were harvested after 48 h or 72 h of transfection for protein or RNA analysis. Western blotting was performed for ALR, TGF-β receptor II (TGFβ-RII), collagen 1A1 (COLL1A1), alpha-smooth muscle cell actin (α-SMA), rac1, E-cadherin and β-actin. Quantitative RT-PCR was performed for ALR, GAPDH, miRNA-181a or 5S rRNA.

**Results:**

TGF-β induced the expression of miRNA-181a, which in turn down-regulated ALR thereby induced the fibrosis markers, such as COLL1A1, α-SMA and rac1 in hepatic stellate cells. Over-expression of miRNA-181a down-regulated expression of ALR and up-regulated expression of fibrosis markers. On the other hand, ALR over-expression resulted in a decrease in miRNA-181a expression and fibrosis markers. Over-expression of ALR also inhibited the expression of TGFβ-RII and increased expression E-cadherin.

**Conclusion:**

TGF-β induced miRNA-181a, which in turn induced fibrosis, at least in part, by inhibiting ALR. ALR inhibited TGF-β action by decreasing the expression of TGFβ-RII, thereby inhibiting miRNA-181a expression and fibrosis markers. ALR could serve as a potential molecule to inhibit liver fibrosis.

## 1. Introduction

Hepatic fibrosis is a result of wound healing response due to the liver injury [1]. Hepatic stellate cells normally present in the parasinusoidal space and synthesize extracellular matrix proteins required for the maintenance of basement membrane. Due to liver injury, secreted soluble factors and change in the stiffness of the matrix, stellate cells are activated and transdifferentiate into myofibroblast-like cells. Once activated, they proliferate and secrete α-smooth muscle cell actin (α-SMA), transforming growth factor-β (TGF-β), platelete derived growth factor (PDGF) and connective tissue growth factor [2]. TGF-β in turn promotes fibrogenesis and epithelial mesenchymal transition [3]. Recent reports showed that miRNAs could play a role in TGF-β mediated liver fibrosis [4, 5]. microRNAs are small non-coding RNAs, which regulate cell functions at post-transcriptional level. It was shown that miRNA-19b could target TGF-β signaling pathway by binding to TGF-β receptor II (TGF-β RII) and inhibited stellate cell activation [6]. Overexpression of miRNA-29b inhibited the primary mouse hepatic stellate cell viability and the expression of α-SMA [7]. miRNA-181a was shown to be upregulated in prostate cancer tumors and was shown to be involved in EMT processes [8]. miRNA-181a was also shown to be involved in regulating lung fibrogenesis [9]. Recently it was shown that miRNA-181a-5p enhanced cell proliferation via regulating TGF-β signaling in medullary thymic epithelial cells [10].

Augmenter of liver regeneration (ALR)is encoded by GFER, and identified from weanling and regenerating rat livers [11]. The native ALR is a 22 kDa protein, and appears to be modified to three forms (36, 38 and 40 kDa). Although ALR was shown to function as sulfhydryl oxidase, cytochrome C reductase, inducer of cytosolic protein Fe/S maturation, and inhibitor of hepatic NK cell cytotoxicity and Kupffer cell activation, the specific function of the three forms of ALR is not known [12]. In cultured hepatocytes, Inhibition of ALR led to mitochondrial dysfunction and cell death. Recently it was shown that liver-specific conditional knockout of ALR in mouse caused an increase in steatosis and apoptosis of hepatocytes within 2 weeks of post-birth [12]. ALR was shown to inhibit liver fibrosis in mice model system [13]. The mechanisms by which it inhibited fibrosis was not known. In this manuscript we showed that TGF-β induced firbogenic markers, at least in part, via miRNA-181a which in turn inhibited ALR. Overexpression of ALR inhibited TGF-β RII expression thereby decreasing TGF-β mediated fibrogenic markers in hepatic stellate cells.

## 2. Materials and methods

### 2.1 Patient samples, cell culture and TGF-β treatment

All the human liver samples were obtained from the liver clinic patients came to Institute of Liver and Biliary Sciences, New Delhi. The samples were obtained after the Institutional human ethical committee approval and after obtaining written consent from the participants. The control samples (n = 5) were obtained from the healthy liver donors who came for liver transplantation and the cirrhotic liver samples were obtained from the liver transplant recipient patients (n = 5). These samples were homogenized and used for total RNA isolation and real-time PCR. Human stellate cell line, LX2, were cultured in Dulbecco’s Minimal Essential Media (DMEM) (HiMedia Laboratories, Mumbai, India) supplemented with 10% fetal bovine serum (Invitrogen, Carlsbad, CA, USA) and 1 mg/ml penicillin and streptomycin (HiMedia Laboratories) at 37°C and 5% CO_2._ After 24 h, the media was washed and TGF-β (20 ng/ml) (R & D Systems, Minneapolis, MN, USA) was added to the cells in serum free DMEM. After 24 h of the incubation, the cells were washed, collected either for protein isolation or for RNA purification.

### 2.2 Transfection expreiments

LX-2 cells were plated 24 h before transfection experiments. LX2 cells were washed and Opti-MEM media (Invitrogen) was added. The cells were transfected with ALR expressing plasmid or empty vector (pcDNA3.1+) using SiPortNeoFx reagent (Invitrogen) according to the manufacturer’s protocol. The media was replaced with complete media after 4 h and again replaced with fresh complete media after 20 h. After 48 h of transfection, the cells were harvested for the isolation of protein or purification of RNA. In miRNA transfection experiments, LX-2 cells were transfected either with miRNA-181a mimic (Sigma Aldrich, St Louis, USA) or NS-miRNA (Sigma Aldrich) using SiPortNeoFx reagent. The media was replenished every 24 hours for 3 days. After that the cells were washed and collected.

### 2.3 Western Blotting

The cells were washed with phosphate buffered saline and they were collected with Mammalian Protein Extraction Reagent (Thermo Scientific, USA) containing1% protease inhibitor cocktail (Thermo Scientific, USA). Bicinchoninic acid colorimetric assay (Thermo) was performed to determine protein concentration. Equal concentration of protein (30–50 μg/well) were run in the SDS-PAGE gels (10–12%) and transferred onto PVDF membranes. The membranes were incubated for 1 h with blocking buffer containing 5% Bovine Serum Albumin (HiMedia Laboratories) in 1X Tris-Buffered Saline (TBS) solution containing 0.1% Tween-20 (1X TBS-T). Membranes were then incubated overnight with specific primary antibodies, such as TGF-β RII, Collagen 1A1 (COLL1A1), ALR, β-actin (Santa Cruz Biotechnology, Dallas, TX, USA), α-SMA (Abcam, Cambridge, UK), E-cadherin, and rac 1 (Cell Signaling Technology, Danvers, MA, USA) at 4°C overnight. After washing 6 times x 5 minutes each with 1X TBS-T, the membranes were incubated with corresponding secondary antibodies for an hour and was eventually visualized using ECL Western blotting substrate. The membranes were developed after exposing to photosensitive films. As a loading control, β-actin was used in all the experiments.

### 2.4 Real Time PCR

TRIzol reagent (1ml) was added directly to the cells or the liver homogenate. The total RNA was purified as per the manufacturer’s instructions. Total RNA was used for cDNA synthesis using Universal cDNA synthesis kit (Exiqon, Vedbaek, Denmark). Real-time PCR was performed for miRNA-181a and normalized with 5S rRNA (Exiqon) using 2x Power Up SYBR Green reagent (Invitrogen) according to manufacturer’s protocol. For RT-PCR experiments, cDNA was synthesized from total RNA (1 μg per reaction) using PrimeScript 1^st^ strand cDNA synthesis kit (Takara Bio, USA). cDNA (50 ng per reaction) was used for the real time PCR reaction along with other required reagents. ALR, TGF-β and β-actin (IDT Technologies) gene fragemnts were amplified using specific primers. Analysis of expression was done using 2^-ΔΔCt^ method.

### 2.5. Statistics analysis

All experiments were performed atleast three times (n = 3) in duplicates. The data were expressed as mean **±** standard deviation. Unpaired Student’s t-test between two groups and the values were considered as statistically significant if p<0.05.

## 3. Results

### 3.1. TGF-β induces miRNA-181a expression and inhibits ALR expression

**Fig 1:**
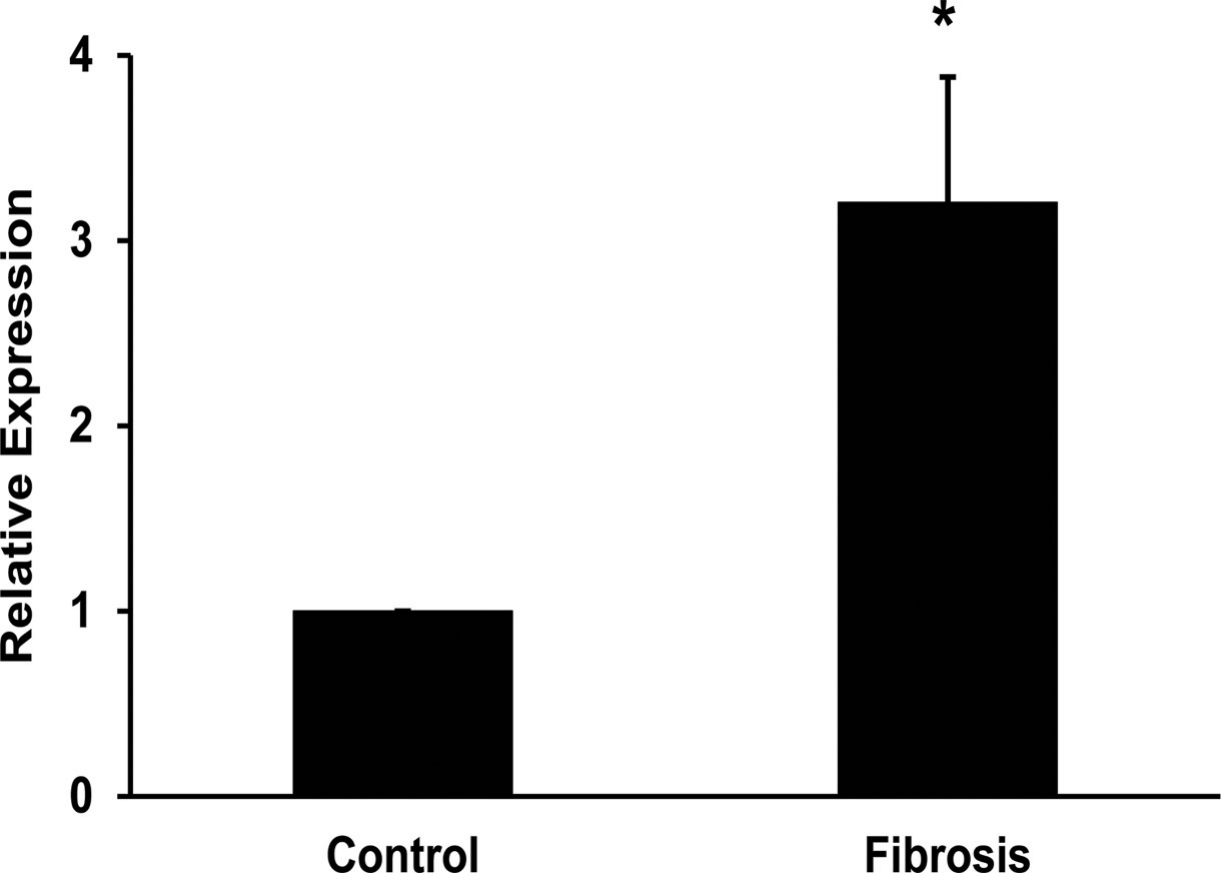
Expression of miRNA-181a in liver tissues. The liver tissues were obtained from the liver transplant participants. Total RNA was purified from the liver tissues of the donors (healthy liver samples) and recipients (cirrhotic patients). cDNA was synthesized and real-time RTPCR was performed to determine the expression of miRNA-181a (n = 5; *p<0.05, compared to healthy controls).

**Fig 2:**
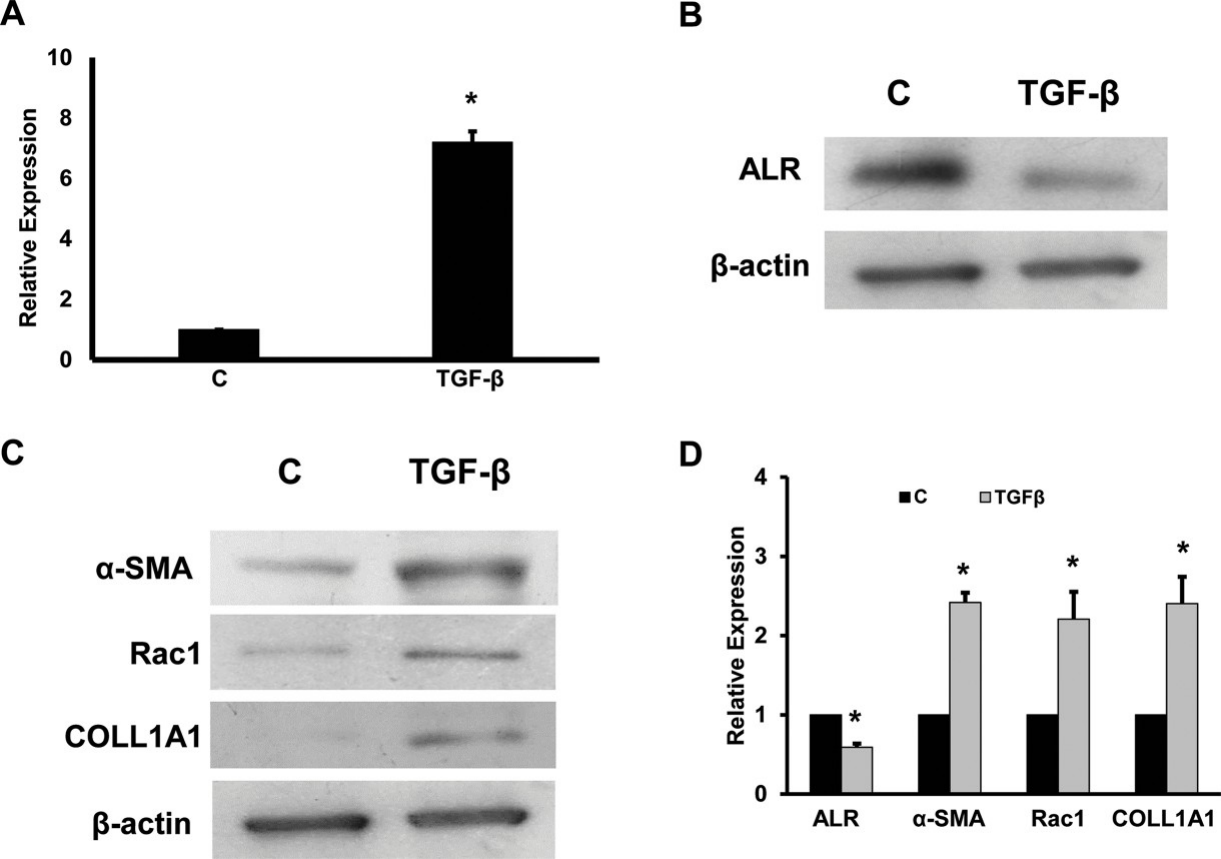
Effect of TGF-β on miRNA-181a, ALR and fibrogenic markers. LX2 cells were incubated with TGF-β for 24 hours under serum-free conditions. **A.** Total RNA was purified and real-time RTPCR was performed for miRNA-181a expression (n = 3; *p<0.05, compared to control). Lane 1, control cells; Lane 2, TGF-β treated cells. **B.** The total cellular protein was isolated and Western blots were run for ALR protein. β-actin was used as an internal control. **C.** Western blots were performed for α-SMA, rac1, COLL1A1 and β-actin. A representative gel picture is shown (n = 3). Lane 1, control cells; and Lane 2, TGF-β treated cells. **D.** The Western blots from 3 experiments were quantitated using Image J software and presented as relative expression (*p<0.05, compared to control).

TGF-β is a profibrogenic cytokine and known to induce fibrosis mainly by activating SMAD signaling pathway. It is also shown to promote epithelial mesenchymal transition (EMT) in chronic liver disease. miRNA-181a was also shown to promote EMT during fibrosis. Hence, it was hypothesized that miRNA-181a might be involved in TGF-β singaling pathway in the stellate cell activation and fibrosis. First, the intracellular expression of miRNA-181a expression was determined in the liver tissues from the healthy liver samples and the cirrhotic liver samples. It was found that there was a significant increase in the expression of miRNA-181a in the cirrhotic liver patients (Fig 1, p<0.05). Incubation of LX-2 cells with TGF-β (20 ng/ml) for 24 hours under serum free conditions resulted in a 7.2-fold increase of miRNA-181a expression as determined by real time RT-PCR (Fig 2A). The Western blots were performed from the total cellular protein and found that ALR protein levels were inhibited by TGF-β treatment (Fig 2B). In all the Western blot experiments β-actin was used as an internal control. To confirm the effect of TGF-β on inducing fibrotic markers, Western blots for α-SMA, rac1 and COLL1A1 was performed. The results showed that there was an increase in the fibrotic markers (Fig 2C). The Western blots results were quantitated using Image J software and found that there was a 2.4-fold, 2.2-fold and 2.4-fold increase of α-SMA, rac1 and COLL1A1 respectively with TGF-β treatment (Fig 2D). There was a 40% inhibition of ALR protein levels with TGF-β treatment (Fig 2D).

### 3.2. miRNA-181a inhibits ALR expression

**Fig 3:**
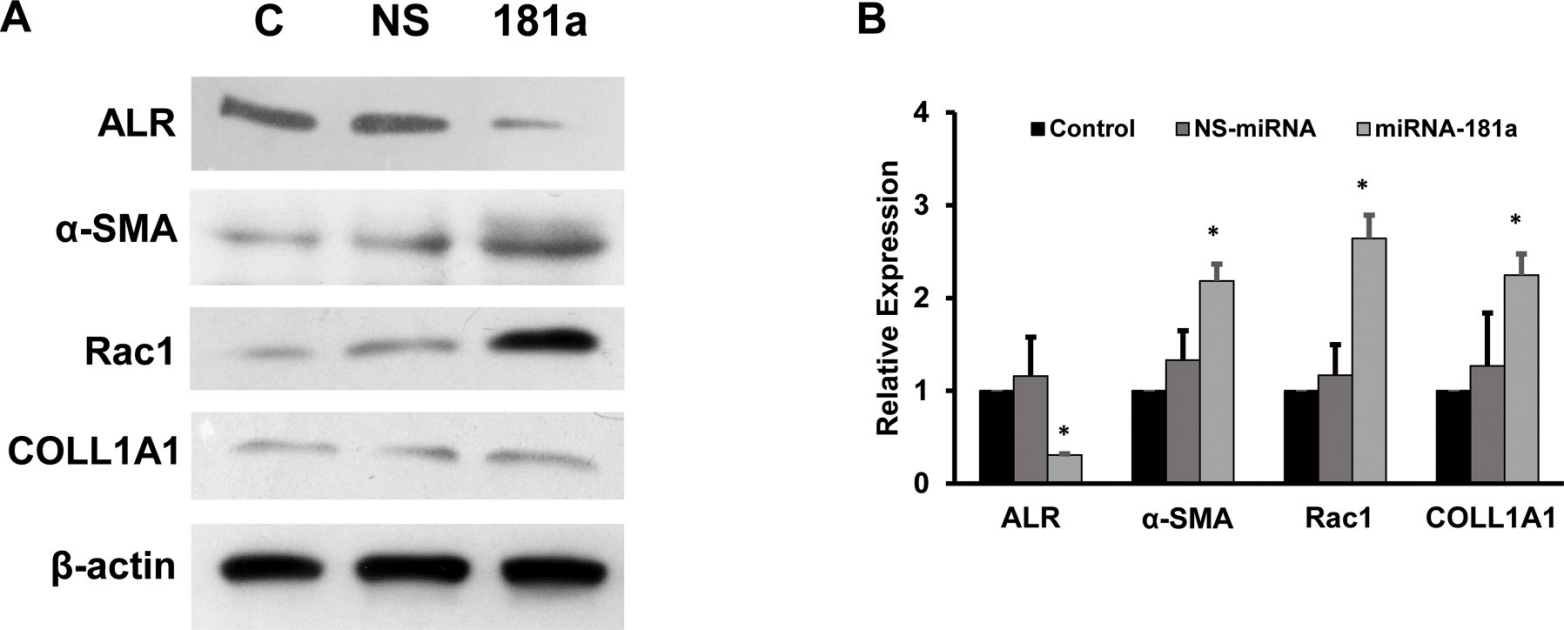
Role of miRNA-181a on the expression of ALR and fibrogenic markers. **A.** LX2 cells were transfected with miRNA-181a mimics as described in materials and methods. The expression of ALR, α-SMA, rac1, COLL1A1 and β-actin were determined using Western blots. A representative picture from 3 experiments is shown. **B.** The bands from the Western blot experiments were quantitated using Image J software and the data are presented as relative expression (p<0.05, compared to control).

To date, there was no data available to show that the regulation between miRNA-181a and ALR. To test whether miRNA-181a could regulate ALR expression, the LX2 cells were transfected with miRNA-181a premiRs for 72 hours as described in the materialsand methods. The total cellular protein was isolated and Western blots for ALR, α-SMA, rac1 and COLL1A1 were preformed. It was found that ALR protein expression was significantly inhibited by miRNA-181a (70% inhibition, compared to control cells) (Fig 3A and 3B). miRNA-181a overexpression resulted in a significant increase in the protein levels of α-SMA, rac1, COLL1A1 (2.2-fold, 2.6-fold and 2.3-fold respectively) (Fig 3A and 3B). These results clearly showed that miRNA could inhibit the ALR expression and increase the fibrogenic markers.

### 3.3. ALR overexpression inhibits miRNA-181a expression and fibrosis markers

**Fig 4:**
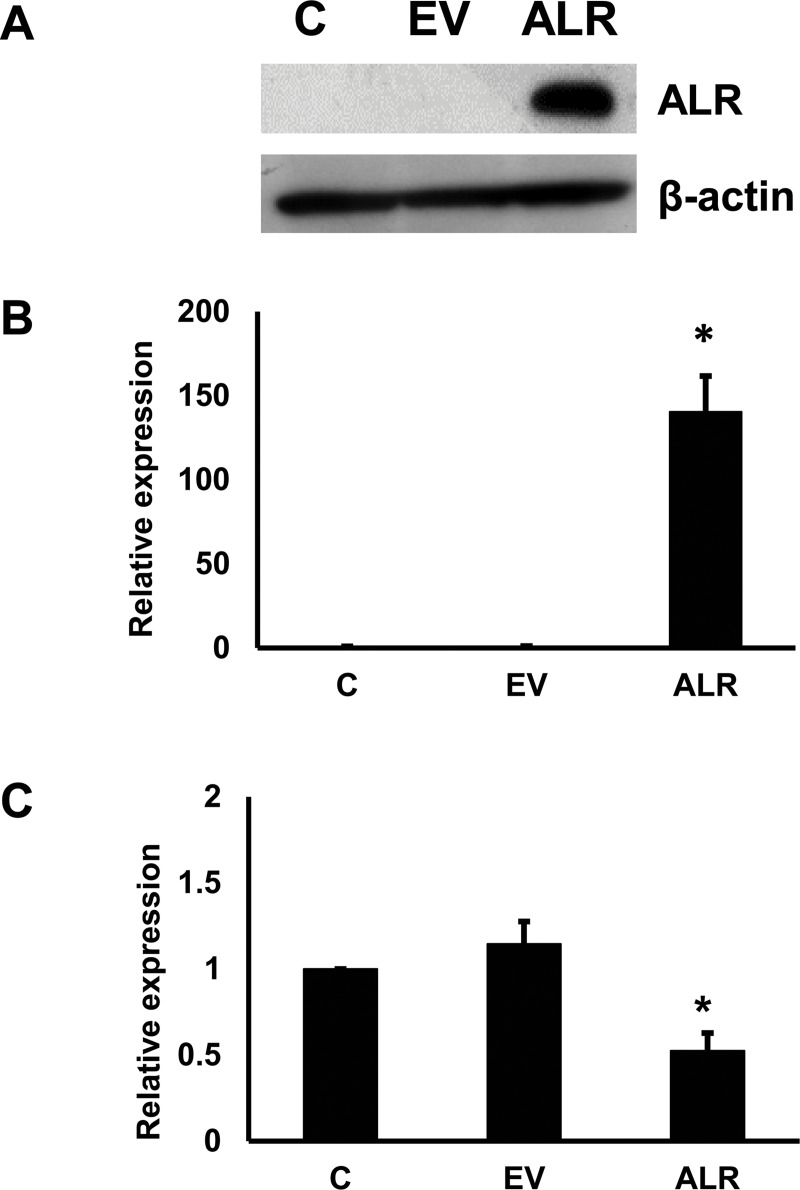
Effect of overexpression of ALR on miRNA-181a expression. ALR was overexpressed in LX2 cells. As a transfection control empty vector was used. C, Control; EV, Empty Vector tranfected cells; and ALR, ALR expressing cells **A.** ALR protein expression was determined in the transfected cells by Western blots (n = 3). **B.** ALR mRNA expression was determined (n = 3; *p<0.05, compared to control). **C.** miRNA-181a expression was determined in ALR expressing cells (n = 3; *p<0.05, compared to control).

Next, whether ALR overexpression could inhibit fibrogenic markers was tested. First, ALR was cloned in pCDNA vector and the expression of ALR was confirmed in LX2 cells. Next, ALR expression plasmid or empty vector were transfected into LX2 cells. After 48 h, ALR expression was determined by Western blots in these cells. There was a high level of ALR protein (Fig 4A) and mRNA expression (Fig 4B) in the cells transfected with plasmid containing ALR compared to empty vector transfected cells. Next, if effect of ALR on miRNA-181a expression was determined. The real time RT-PCR experiments showed that ALR overexpression significantly inhibited miRNA-181a expression by 48% (Fig 4C).

### 3.4.ALR targets TGF β-RII expression

**Fig 5:**
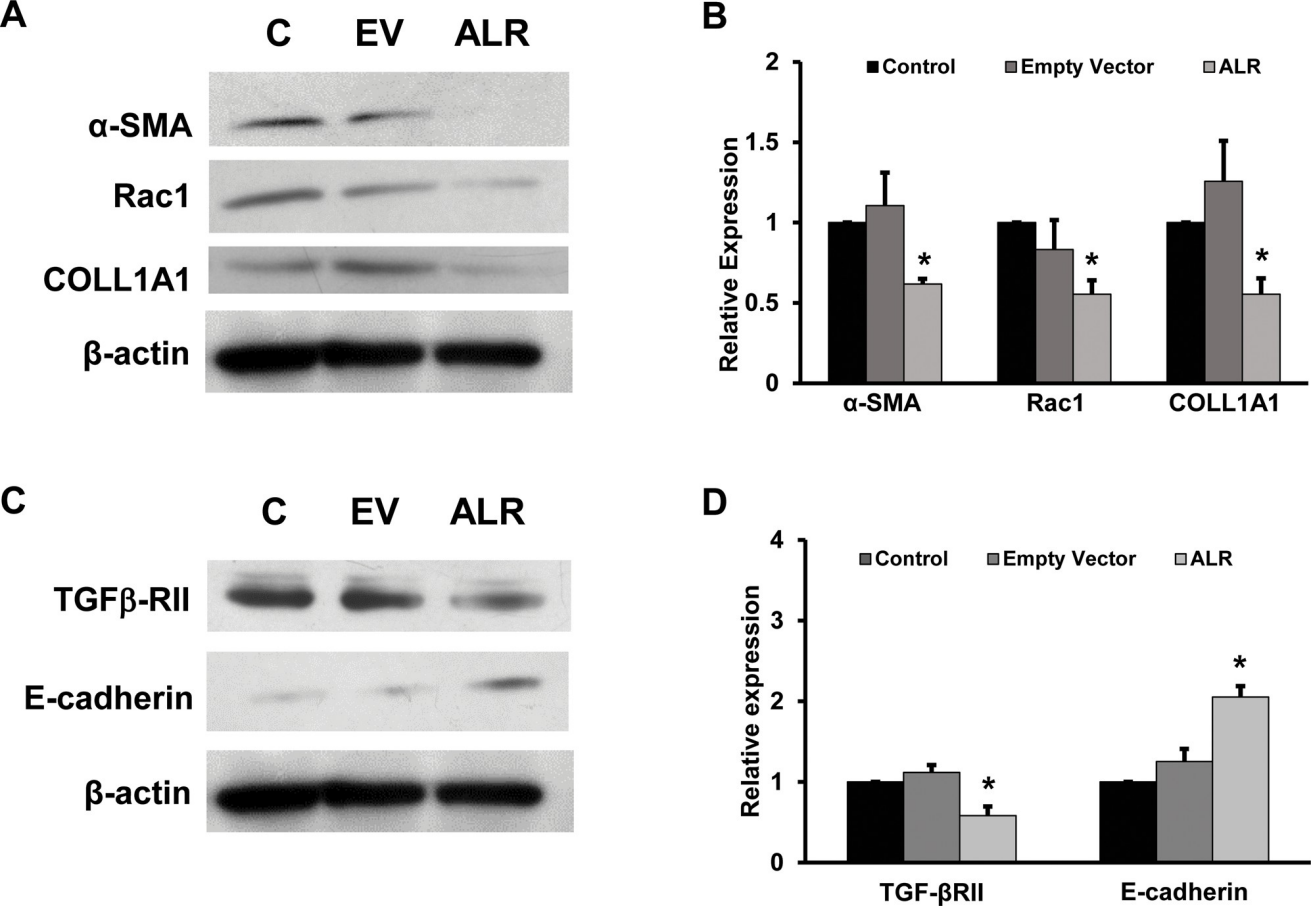
Effect of ALR on fibrogenic markers, TGF-β RII and E-Cadherin. The total cellular protein was isolated from ALR overexpressing cells and Western blots were performed. C, Control; EV, Empty Vector tranfected cells; and ALR, ALR expressing cells. **A.** The representative picture shows the Western blot results of α-SMA, rac1, COLL1A1 and β-actin (n = 3). **B.** The Western blots from 3 experiments of the firbogenic markers were quantitated using Image J software and presented as relative expression (*p<0.05, compared to control). **C.** Western blots were performed to determined the expression of TGF-β RII and E-cadherin. **D.** The band intensities of TGF-β RII and E-cadherin was quantitated by Image J software and presented as relative expression (n = 3; *p<0.05, compared to control).

To determine the link between ALR and miRNA-181a expression, the cells were transfected with ALR expression plasmid and the receptor for TGF-β was determined. It was found that ALR overexpression resulted in an inhibition of TGFβ-RII expression (Fig 5A). Finally the role of ALR in inhibiting fibrosis markers were determined. It was found that ALR overexpression significantly inhibited the expression of α-SMA, rac1 and COLL1A1 as determined by Western blots (Fig 5A). Since ALR decreased miRNA-181a, it was hypothesized that ALR might induce the epithelial marker, E-cadherin. When the cells were transfected with ALR plasmid, there was an increase in the expression of E-cadherin (Fig 5A). The fibrosis markers were quantitated using Image J software and found that there was a 40%, 45% and 45% inhibition of the expression of α-SMA, rac1 and COLL1A1 proteins respectively (Fig 5B).

## 4. Discussion

Several researchers had shown that TGF-β induced fibrosis via activating RSMADs [14, 15]. TGF-β-induced SMADs were shown to induce miRNA-29 and miRNA-200, whereas inhibited miRNA-21 and miRNA-192, thereby inducing renal fibrosis [14]. There are no reports whether TGF-β induced liver fibrosis via miRNA-181a. For the first time we showed that TGF-β induced miRNA-181a, which in turn enhanced fibrosis and inhibited ALR expression in stellate cells. In the present study, we also showed that ALR inhibited TGF-β induced fibrosis and induced epithelial marker, E-cadherin, in hepatic stellate cells. Recently, it was shown that miRNA-181a was elevated in myocardial infarction in a rat model [16] and resulted in enhanced fibrosis markers. Our data showed that incubation of TGF-β with LX2 cells increased miRNA-181a expression and inhibition of ALR. Our data is in agreement with the previous data that incubation of cells with TGF-β resulted in increased profibrogenic markers, such as α-SMA, COLL1AI and rac1 [17]. The decrease in the expression of ALR with TGF-β suggested that TGF-β might be enhancing fibrosis, at least in part, by inhibiting ALR. This was well correlated with the previous finding that overexpression of ALR inhibited fibrosis [12].

miRNA-181a was shown to enhance proliferation of renal cell carcinoma [18]. Recently, it was also shown that downregulation of miRNA-181a protected mice from LPS-induced lung injury [19]. miRNA-181a was shown to enhance epithelial mesenchymal transition in lung adenocarcinoma cells [20] and prostate cancer cells [8]. Since several recent studies suggested miRNA-181a had a role in proliferation, fibrosis and EMT, and TGF-β is known to induce fibrosis and EMT, we tested the relationship between miRNA-181a and ALR. Overexpression of miRNA-181a induced fibrosis markers in our system. This was well correlated with a recent study that overexpression of miRNA-181a resulted in enhanced fibrosis markers [16]. miRNA-125b was found to promote stellate cell activation [4]. Similarly, we found that miRNA-181a induced the fibrosis markers and inhibited ALR expression. Although miRNA-181a did not have a seed sequence in ALR mRNA, ALR protein expression was decreased. It might be possible that miRNA-181a might be acting as direct positive regulator of fibrosis by enhancing fibrosis markers, as showed by others and it also might be enhancing fibrosis by inhibiting ALR. Further studies are needed to elucidate the mechanisms by which miRNA-181 could regulate ALR expression.

ALR is critical for mammalian embryonic development. It was found that depletion of hepatic ALR shortly after birth led to a severe steatosis, mitochondrial degeneration and apoptosis of hepatocytes within two weeks, followed by the cell death and regeneration leading to hepatocellular carcinoma [21]. Prviously, it was shown that there was a low levels of hepatic ALR in cirrhotic alcoholic liver disease patients compared with healthy controls [12]. Kumar et al., had shown that hpeatic deficiency of ALR enhanced alcohol-induced liver injury and promoted liver fibrosis in mice model system [13]. Previous studies showed that ALR could inhibit fibrosis in both in vitro and in vivo. As expected, ALR overexpression inhibited expression of fibrosis markers. Surprisingly, it was also found that ALR inhibited miRNA-181a expression.

**Fig 6:**
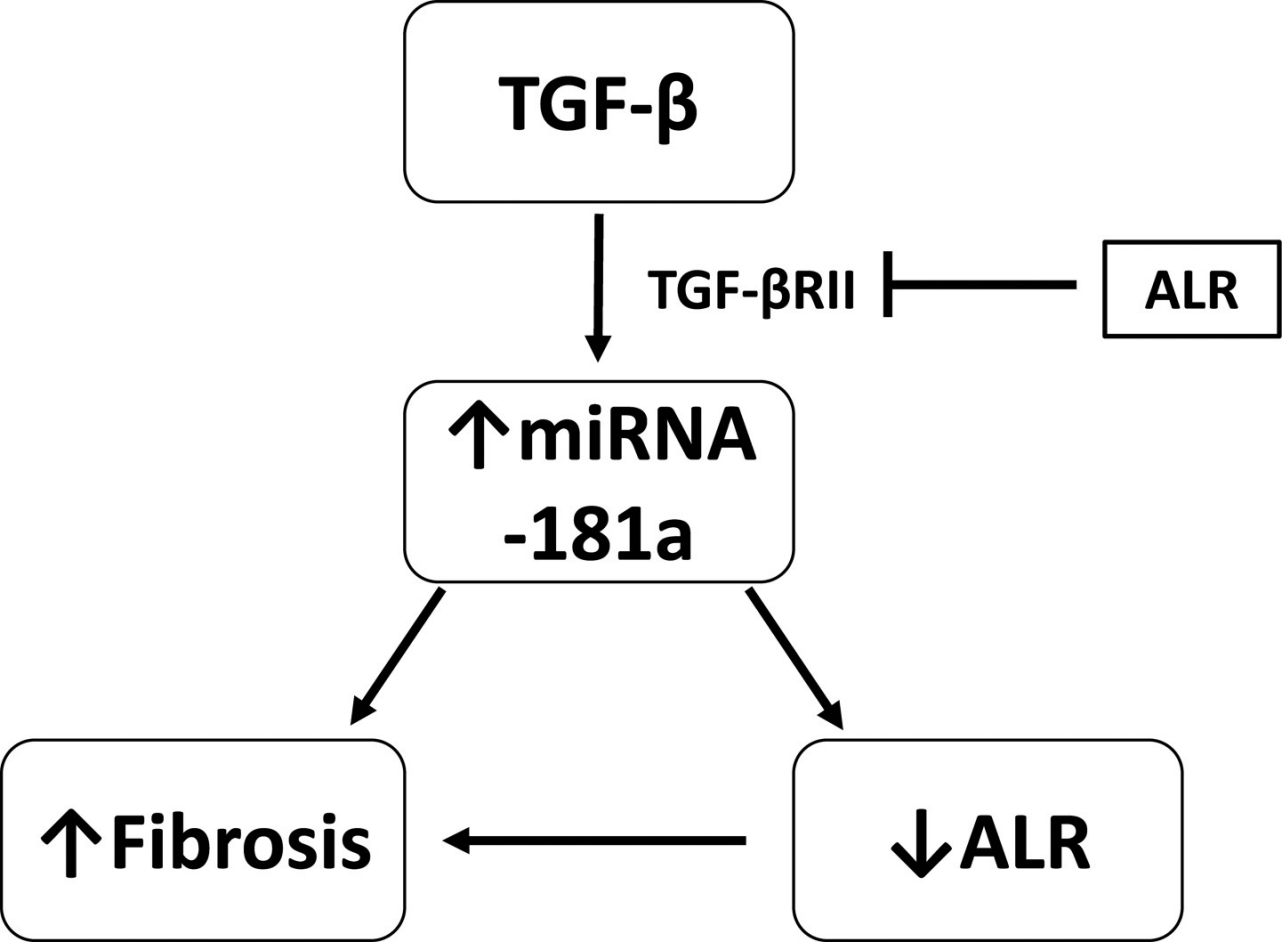
Proposed mechanism of TGF-β-induced miRNA-181a and fibrosis. TGF-β induces fibrosis by upregulating miRNA-181a expression, which in turn inhibits ALR expression and fibrosis. Overexpression of ALR inhibited TGF-β-induced firbrogenic changes by inhibiting TGF-βRII expression.

Previously it was shown that TGF-β/SMAD signaling could play an important role in enhancing renal fibrosis [14, 22]. TGF-β binds to TGF-β RII to activate TGF-β RI and phosphorylates down-stream receptor-associated SMADs, including SMAD2 and SMAD 3 [23]. To demonstrate the role of ALR on the TGF-β activity, effect of ALR on intracellular expression of TGF-β RII was studied. The results showed that there was a significant inhibition of TGF-β RII expression, suggesting that ALR could inhibit all the downstream effects of TGF-β. Next, the effect of ALR on the expression of miRNA-181a, since it was one of the downstream targets of TGF-β. Our results confirmed that the expression of both miRNA-181a and fibrosis markers was inhibited. TGF-β is known to induce EMT transition during chronic liver injury. As expected TGF-β inhibited E-cadherin expression, whereas ALR overexpression enhanced the E-cadherin expression, suggesting that ALR could successfully inhibit TGF-β induced changes in stellate cells.These results clearly showed that ALR, by inhibiting the receptor to TGF-β, inhibited fibrosis and EMT. The proposed mechanism is presented in Fig 6.

In this study we clearly showed that TGF-β induced fibrosis, at least in part, via upregulating miRNA-181a expression and inhibiting ALR expression. Overexpression of ALR inhibited TGF-β-induced firbrogenic changes including the expression of miRNA-181a and E-cadherin.

## Supporting information

S1 Dataset S1 dataset. (ZIP)

## Abbreviations

ALR: Augmenter of liver regeneration
TGF-β: Transforming growth factor-beta
TGF-β RII: Transforming growth factor-beta receptor II
α-SMA: β-smooth muscle cell actin
