# Supplementary figures and images for: TGF-β induces liver fibrosis via miRNA-181a-mediated down regulation of augmenter of liver regeneration in hepatic stellate cells
Source: PLoS One. 2019 Jun 5;14(6):e0214534. doi: 10.1371/journal.pone.0214534 (PMC6550375; doi:10.1371/journal.pone.0214534)

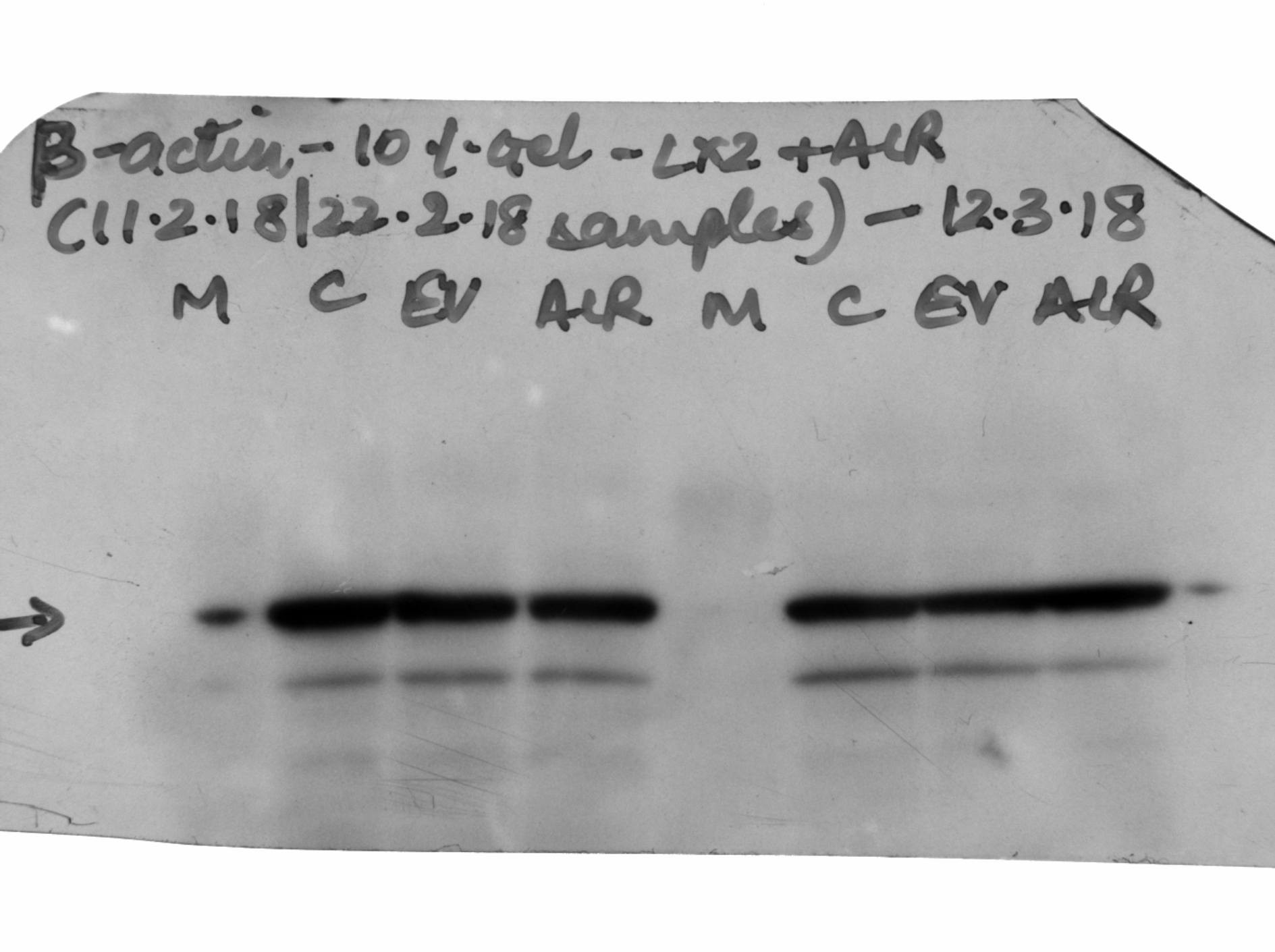

Supplement: S1 Dataset — (ZIP) [file pone.0214534.s001.zip › S1 dataset/Fig.4a_b-actin.tif]

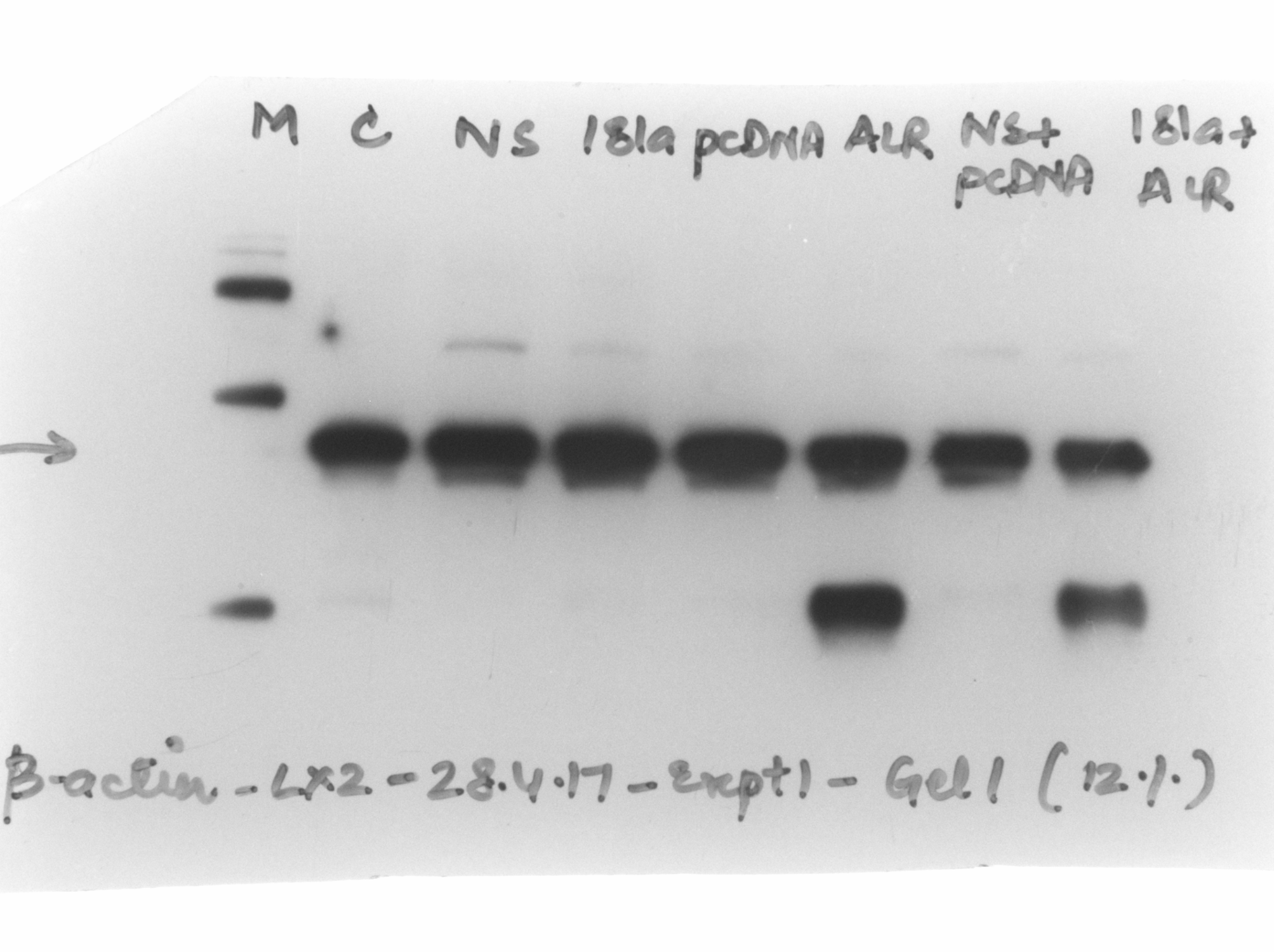

Supplement: S1 Dataset — (ZIP) [file pone.0214534.s001.zip › S1 dataset/Fig.3a_b-actin.tif]

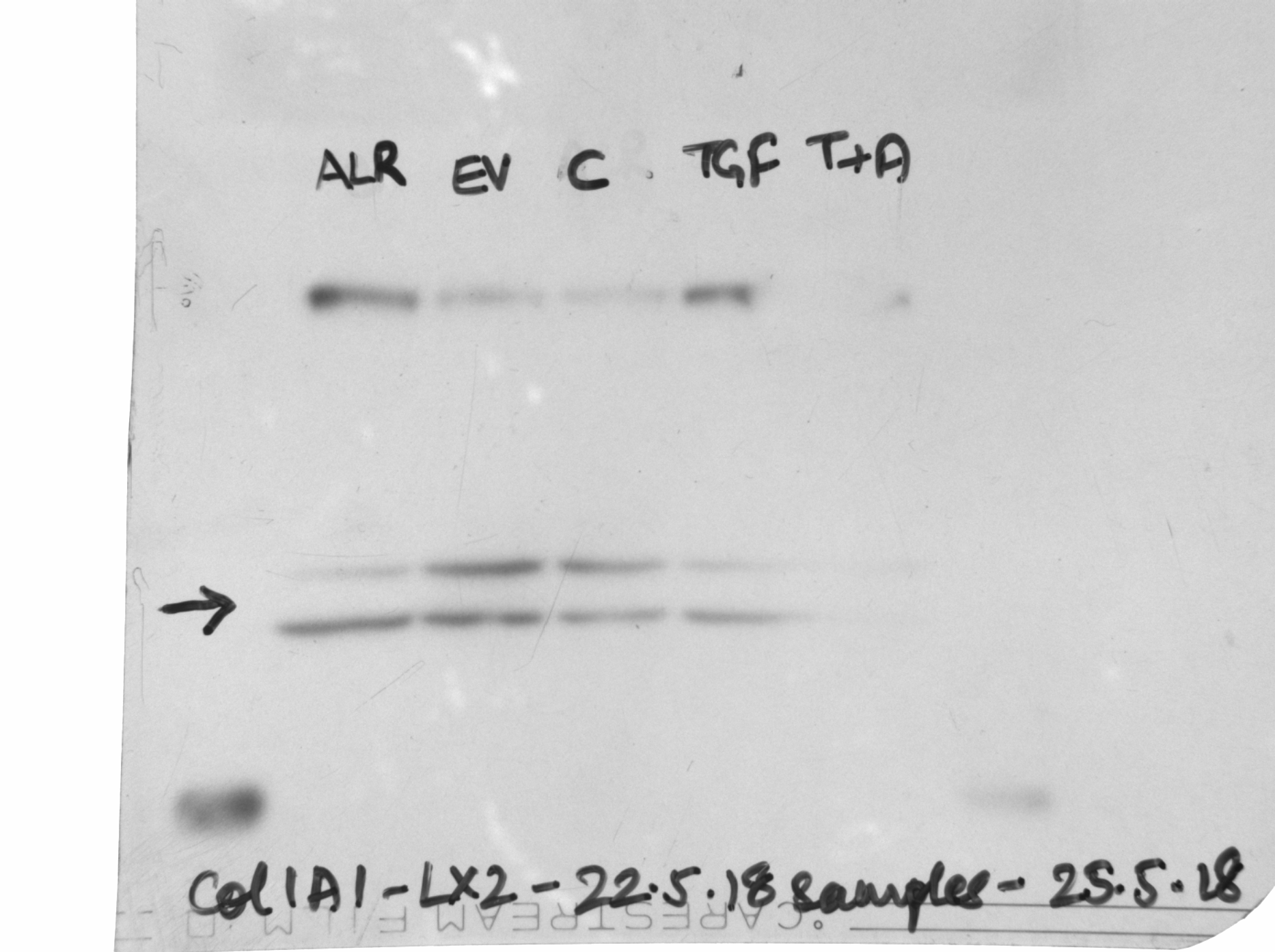

Supplement: S1 Dataset — (ZIP) [file pone.0214534.s001.zip › S1 dataset/Fig.5a_col1a1.tif]

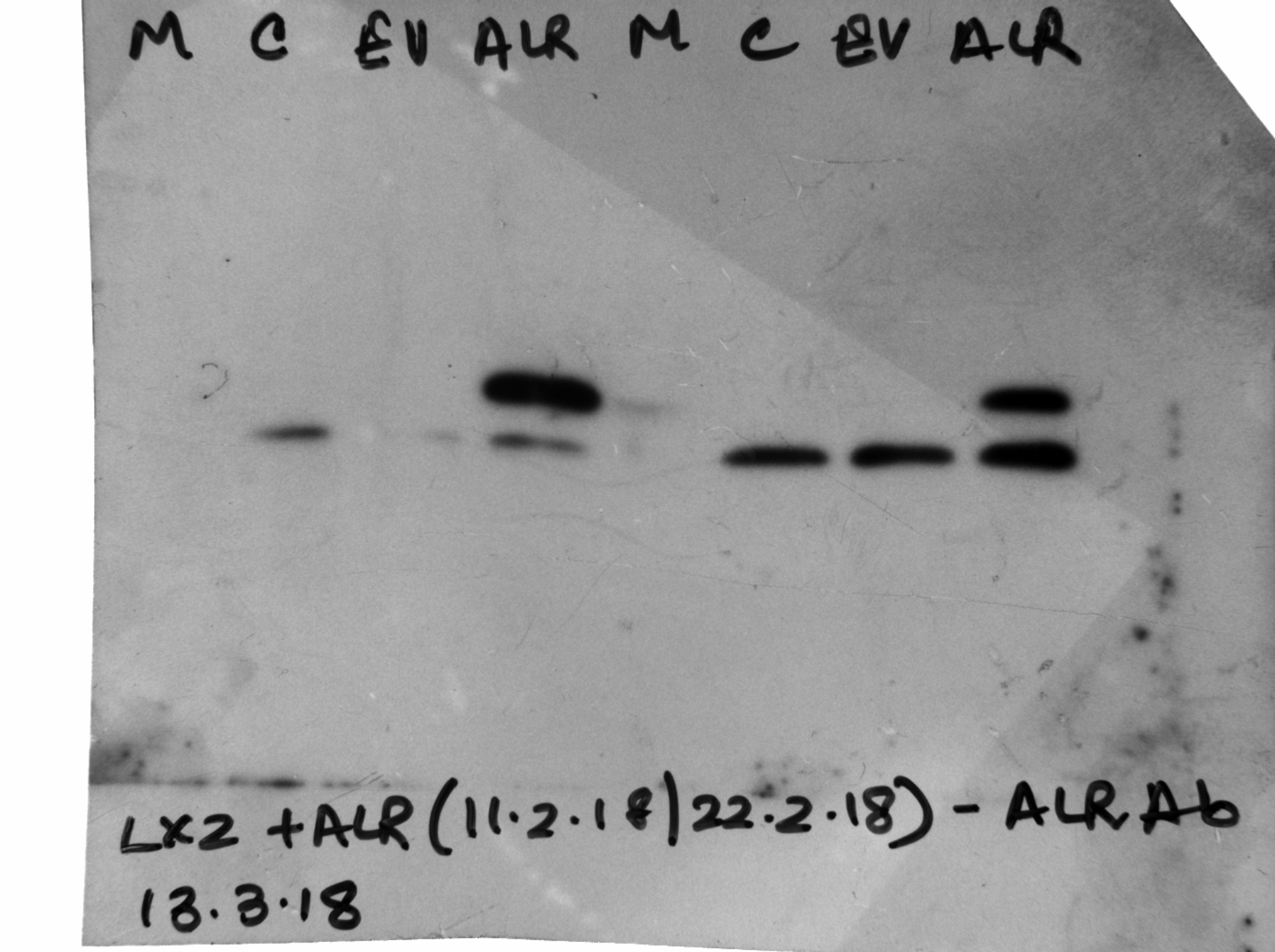

Supplement: S1 Dataset — (ZIP) [file pone.0214534.s001.zip › S1 dataset/Fig.4a_ALR.tif]

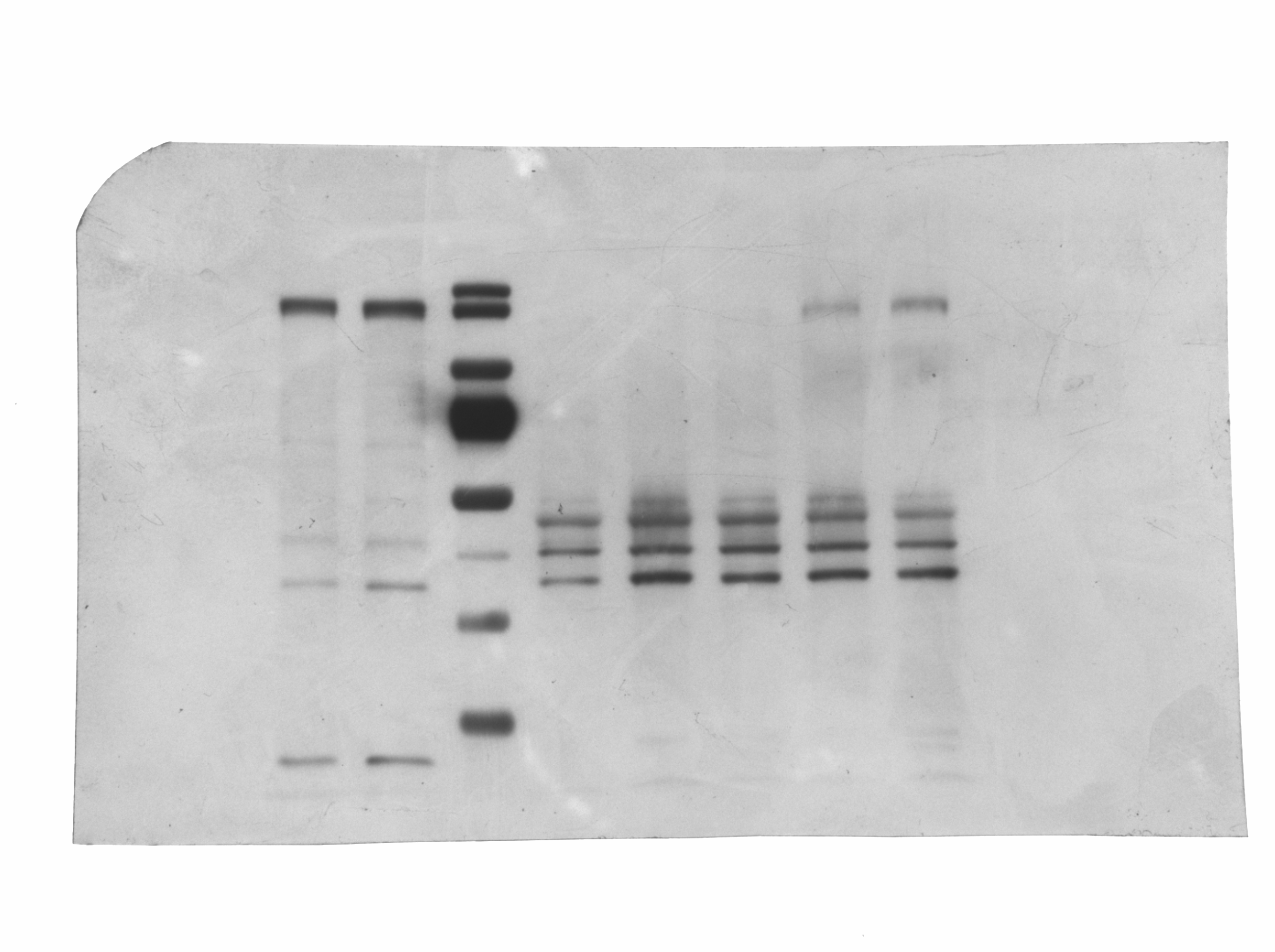

Supplement: S1 Dataset — (ZIP) [file pone.0214534.s001.zip › S1 dataset/Fig.2c_rac1.tif]

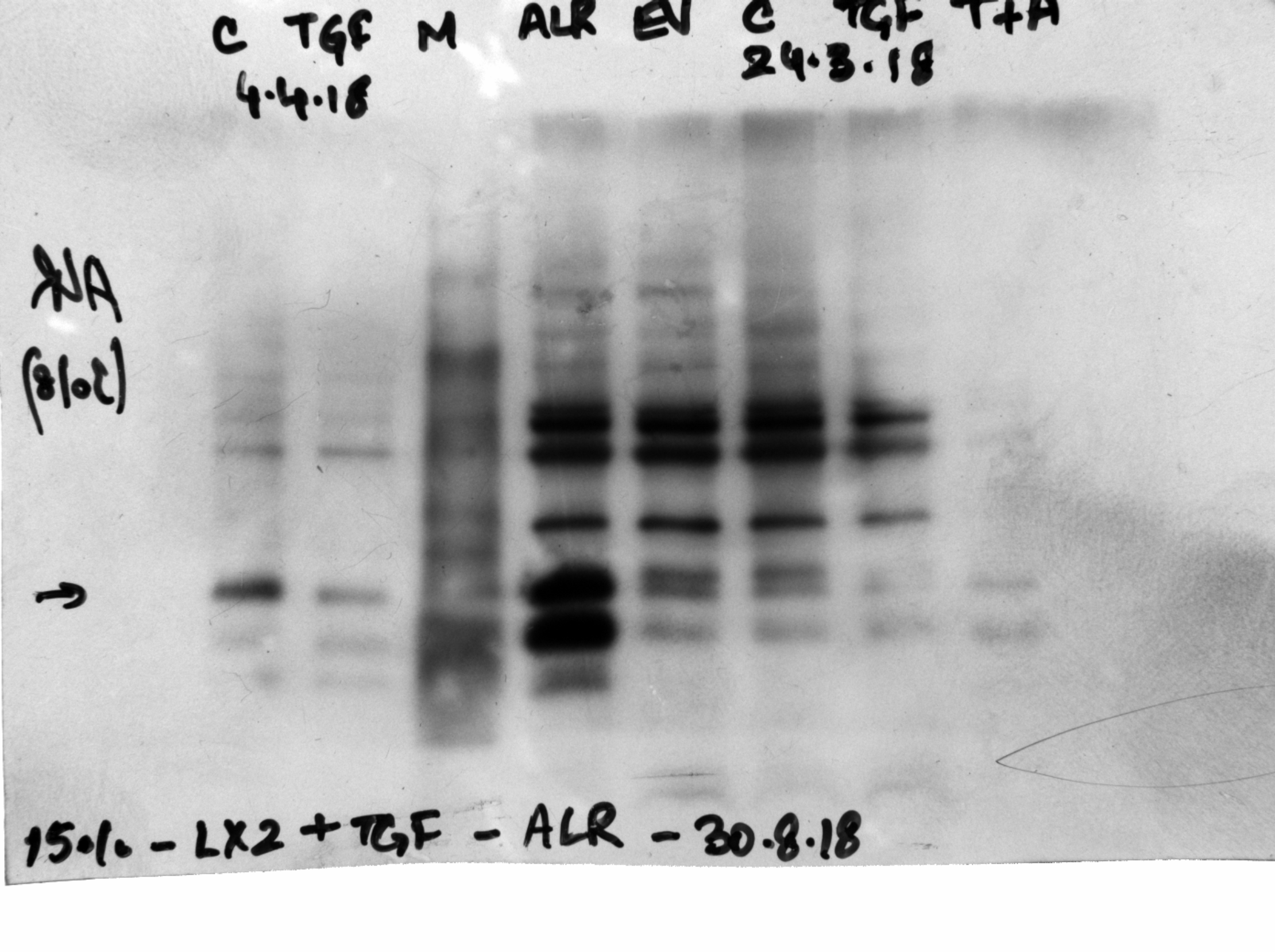

Supplement: S1 Dataset — (ZIP) [file pone.0214534.s001.zip › S1 dataset/Fig.2b_ALR.tif]

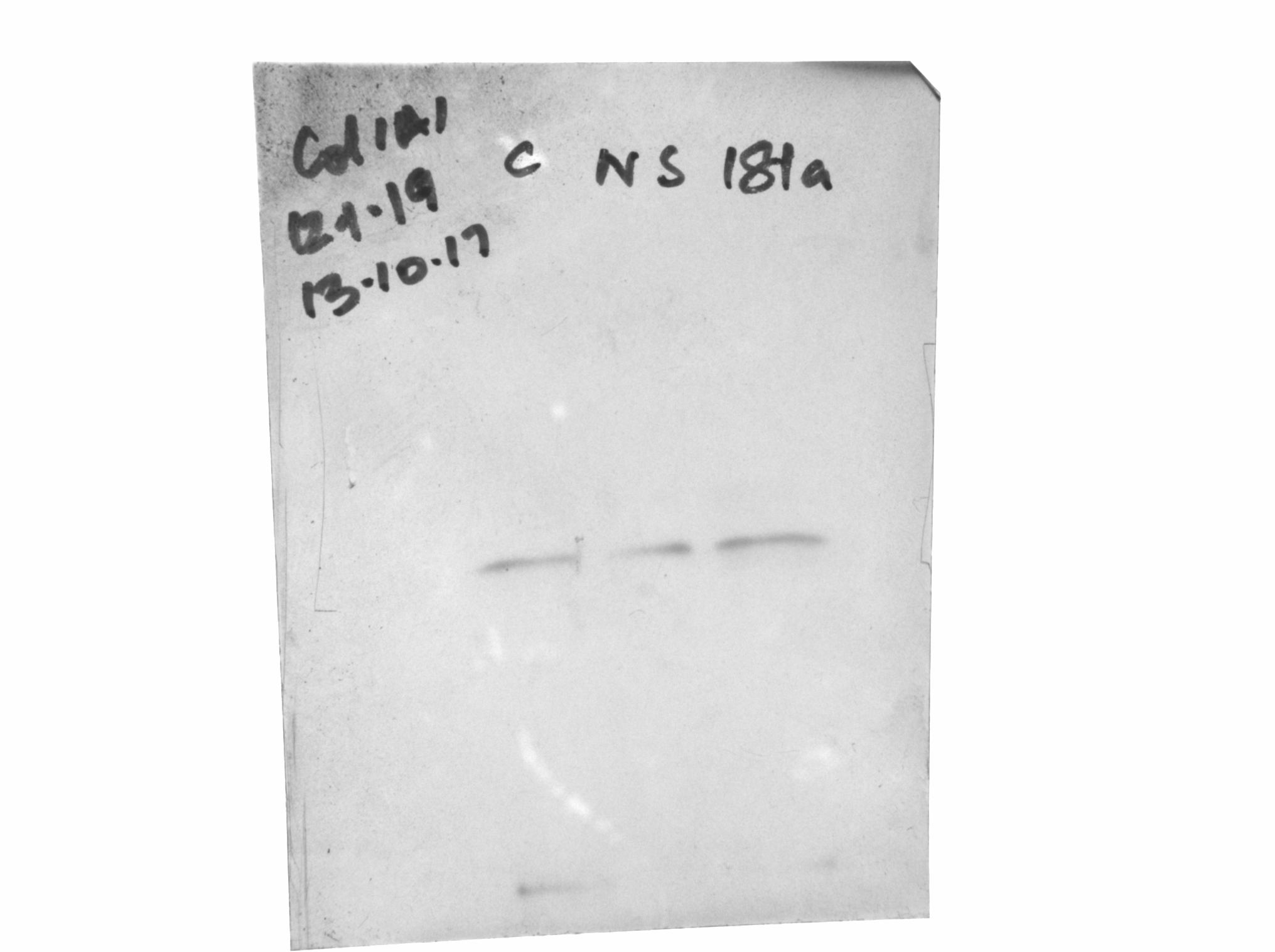

Supplement: S1 Dataset — (ZIP) [file pone.0214534.s001.zip › S1 dataset/Fig.3a_col1a1.tif]

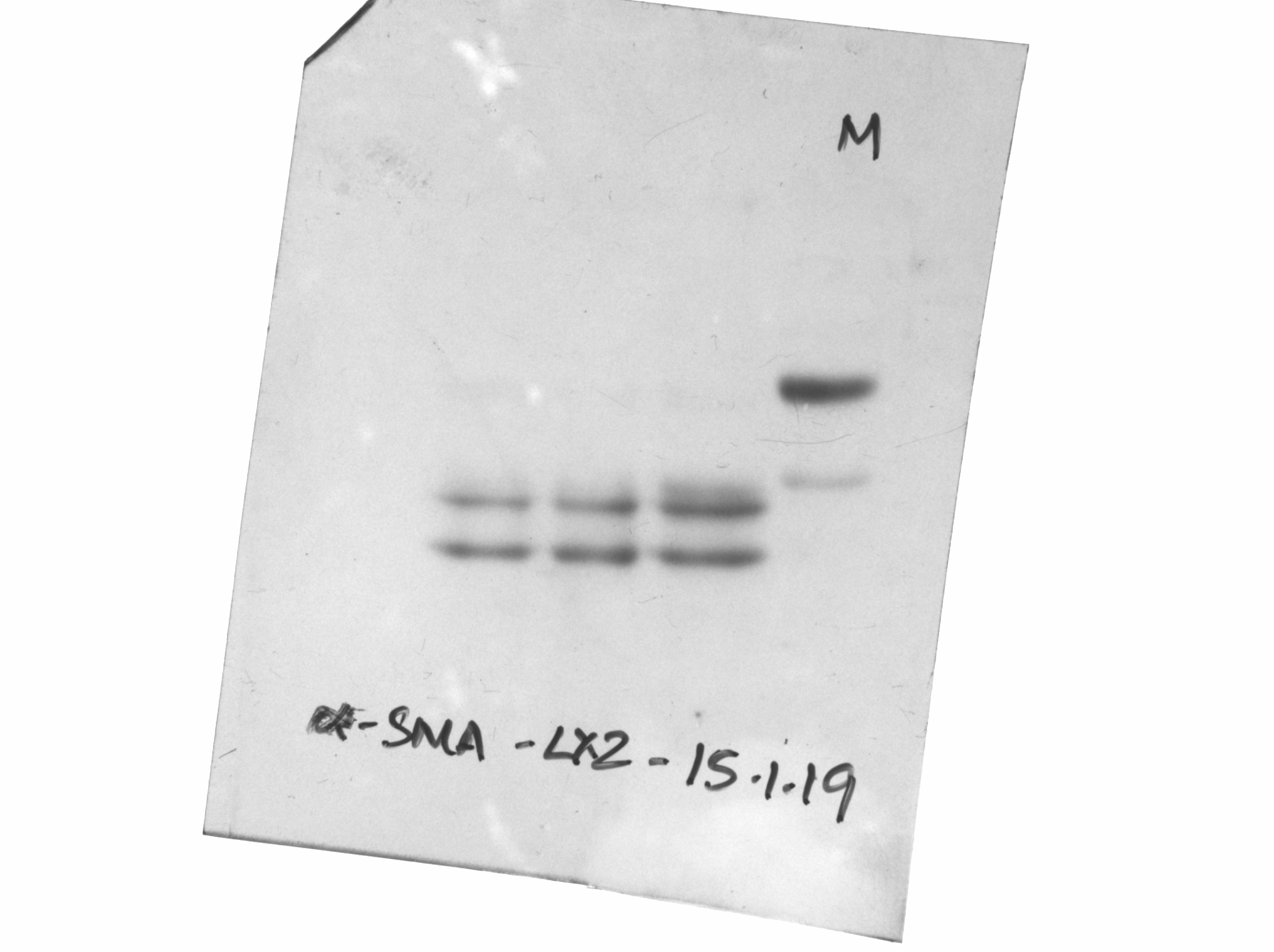

Supplement: S1 Dataset — (ZIP) [file pone.0214534.s001.zip › S1 dataset/Fig.3a_a-sma.tif]

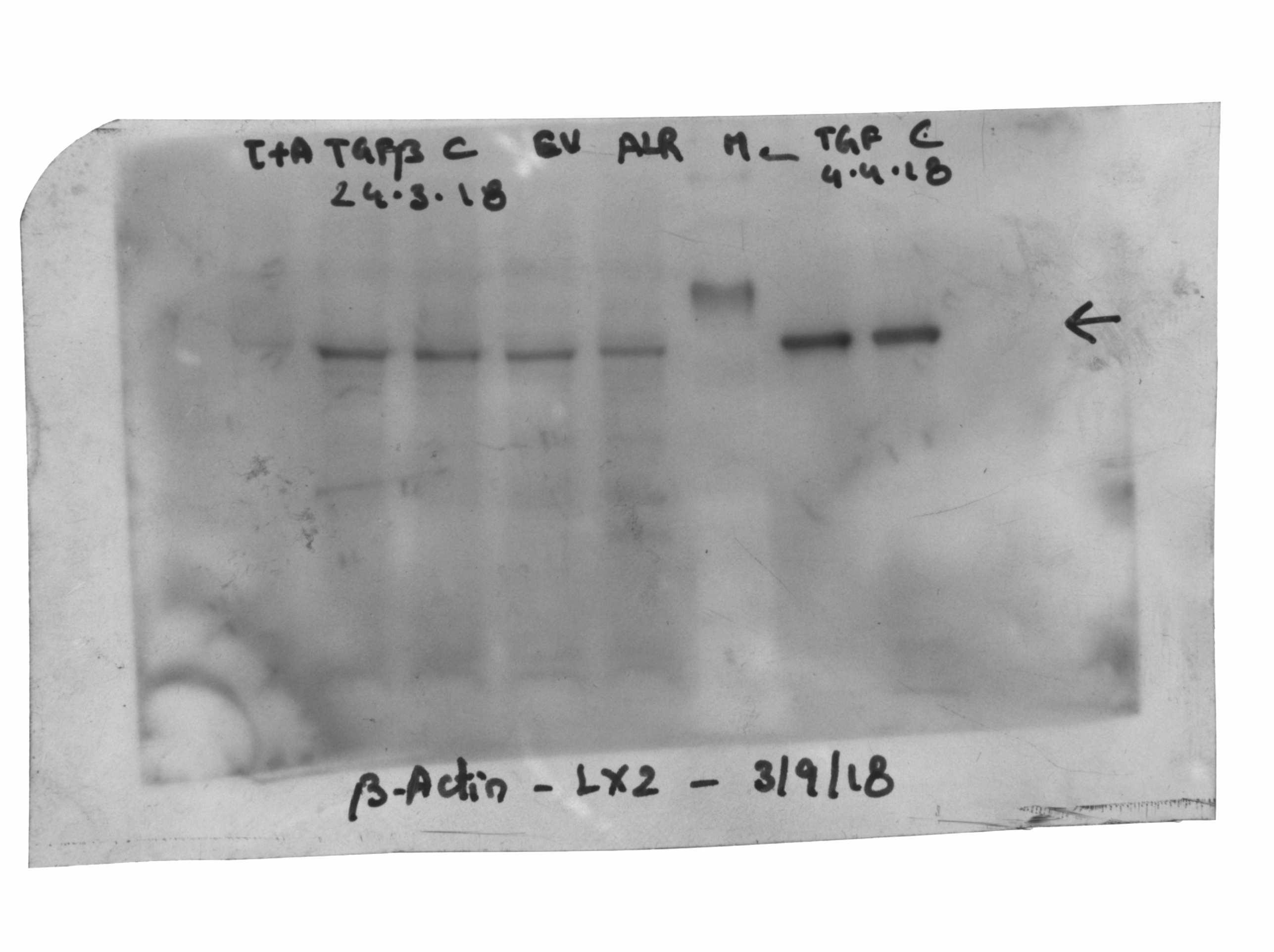

Supplement: S1 Dataset — (ZIP) [file pone.0214534.s001.zip › S1 dataset/Fig.2b_b-actin.tif]

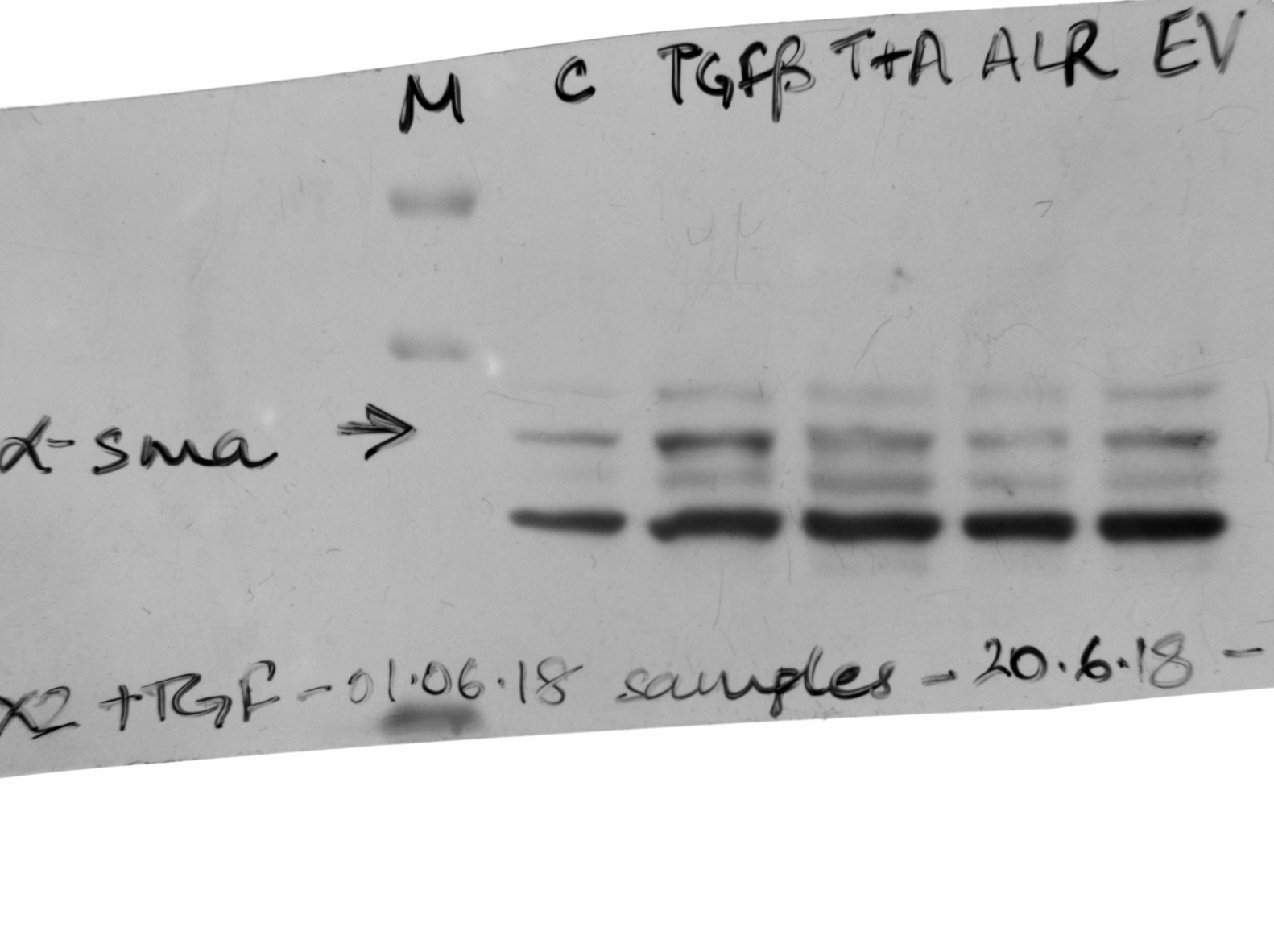

Supplement: S1 Dataset — (ZIP) [file pone.0214534.s001.zip › S1 dataset/Fig.2c_a-sma.tif]

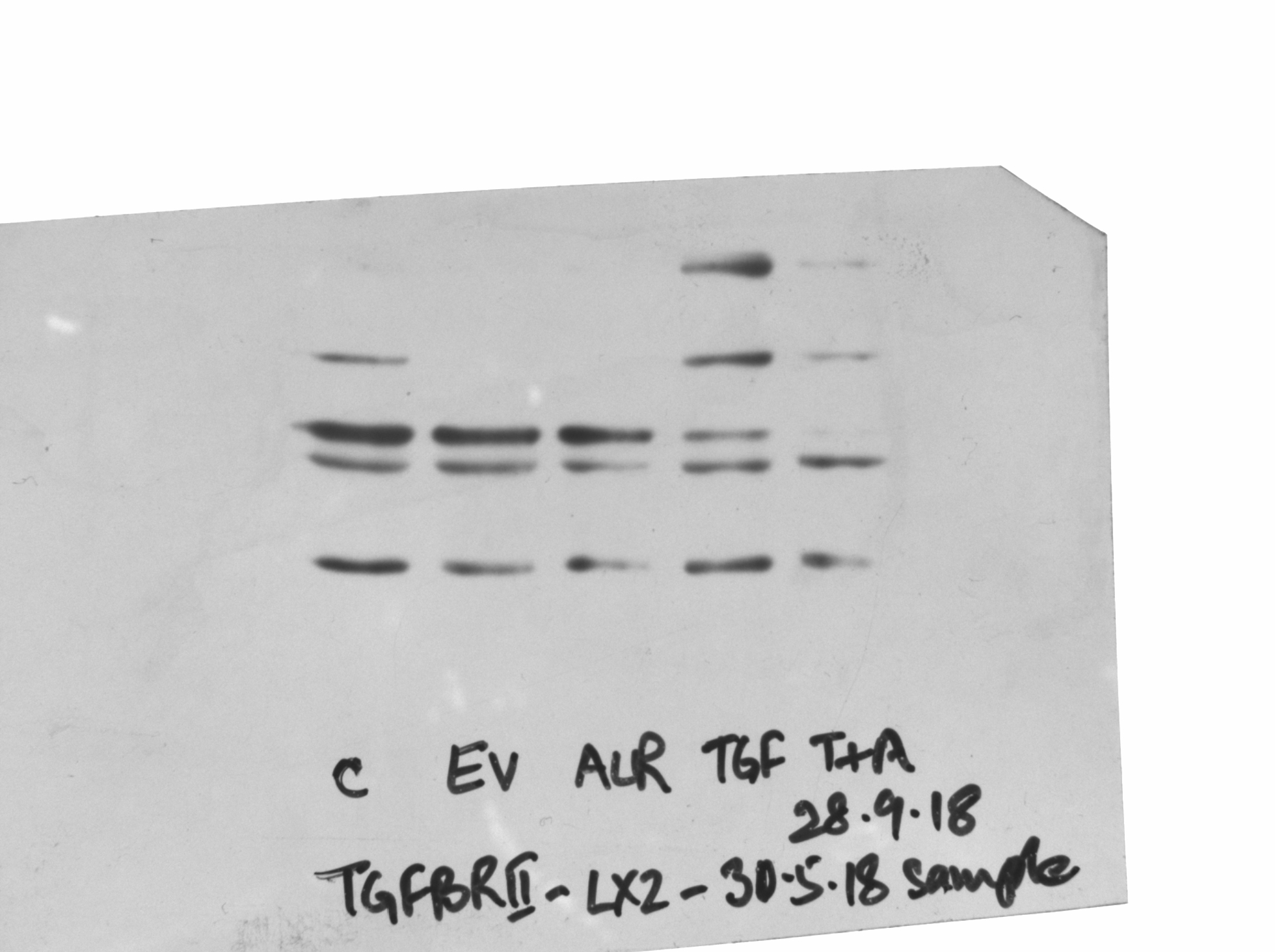

Supplement: S1 Dataset — (ZIP) [file pone.0214534.s001.zip › S1 dataset/Fig.5c_TGFbR_lower bands.jpg]

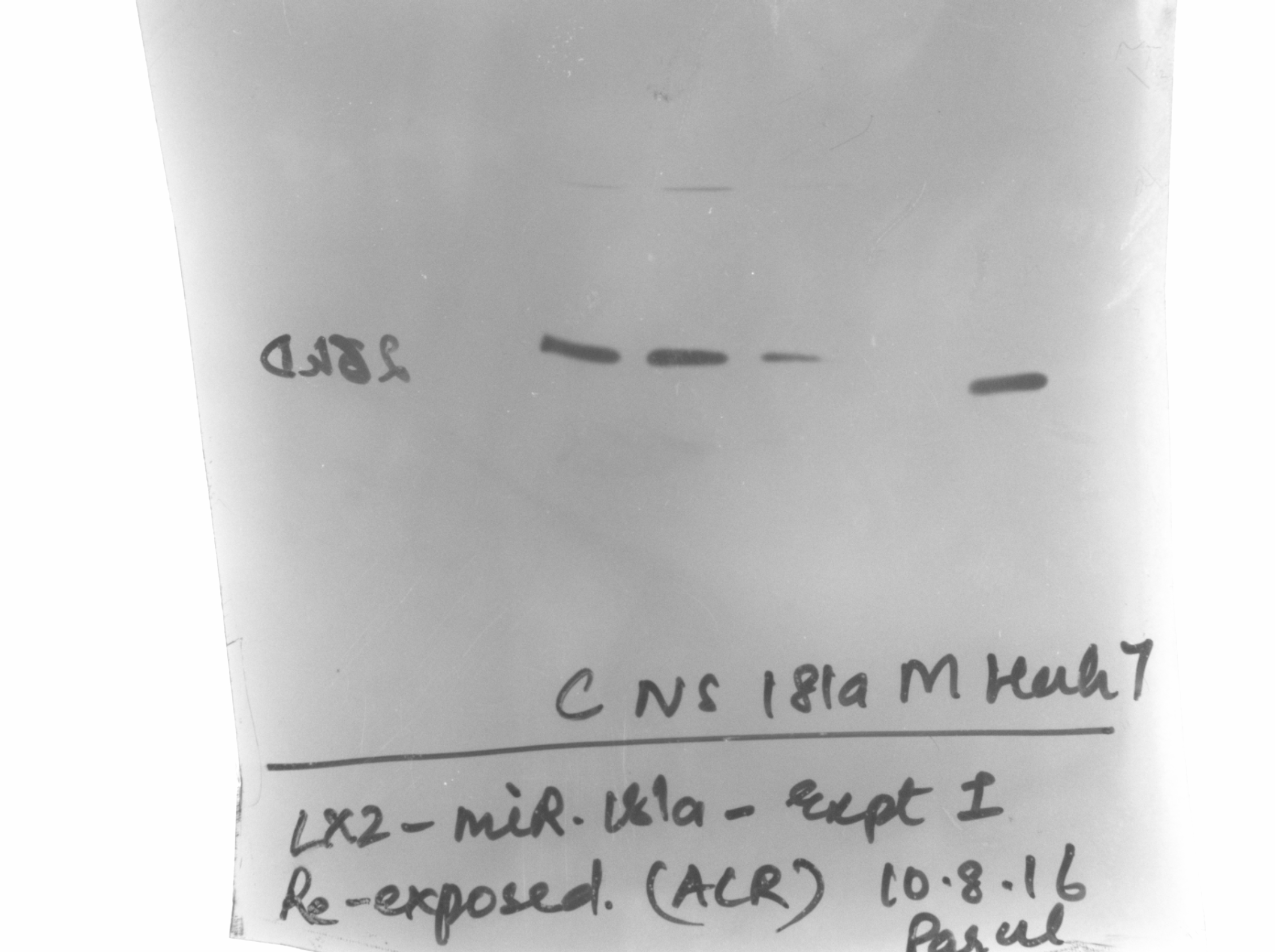

Supplement: S1 Dataset — (ZIP) [file pone.0214534.s001.zip › S1 dataset/Fig.3a_ALR.tif]

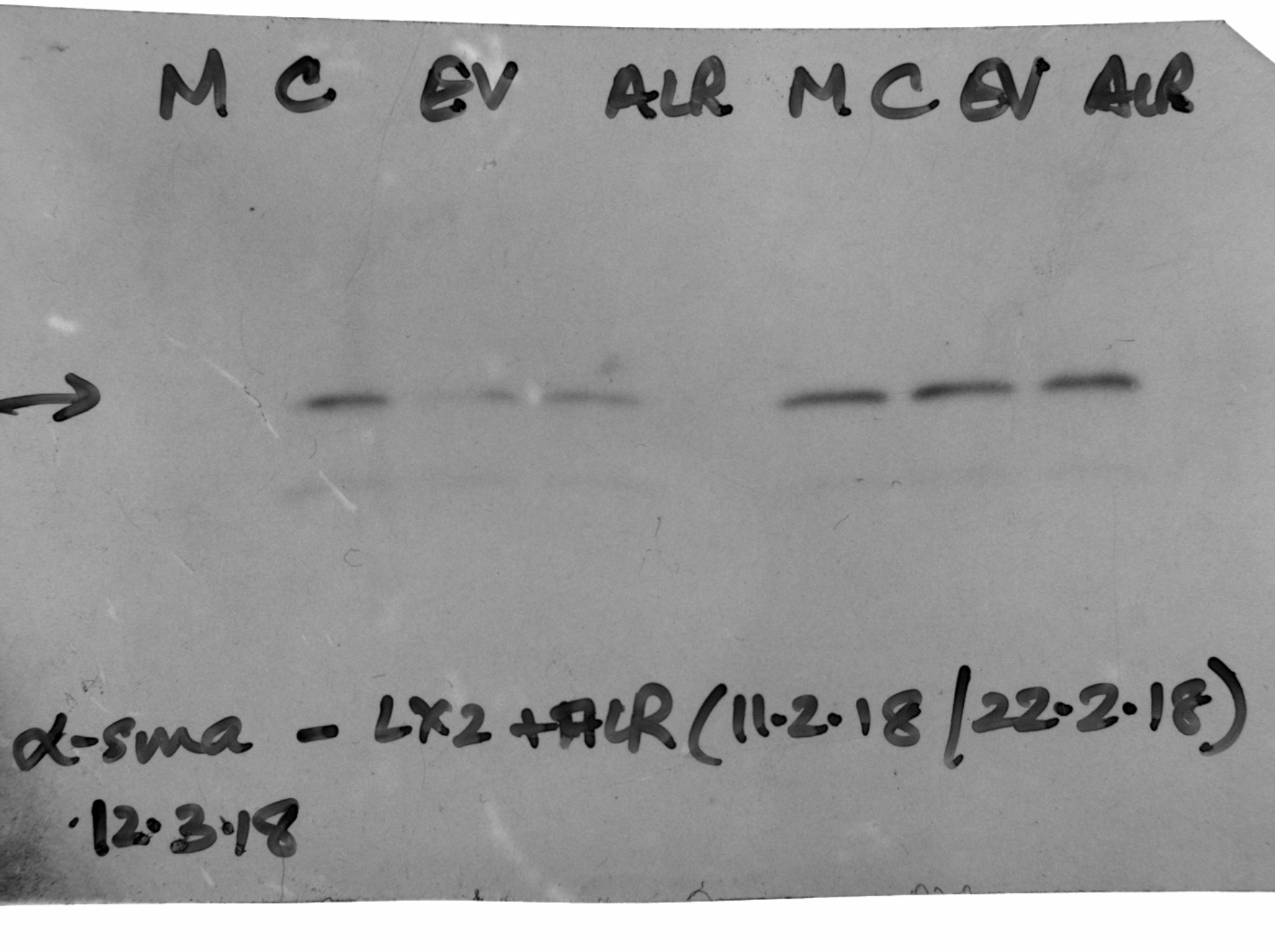

Supplement: S1 Dataset — (ZIP) [file pone.0214534.s001.zip › S1 dataset/Fig.5a_a-sma.tif]

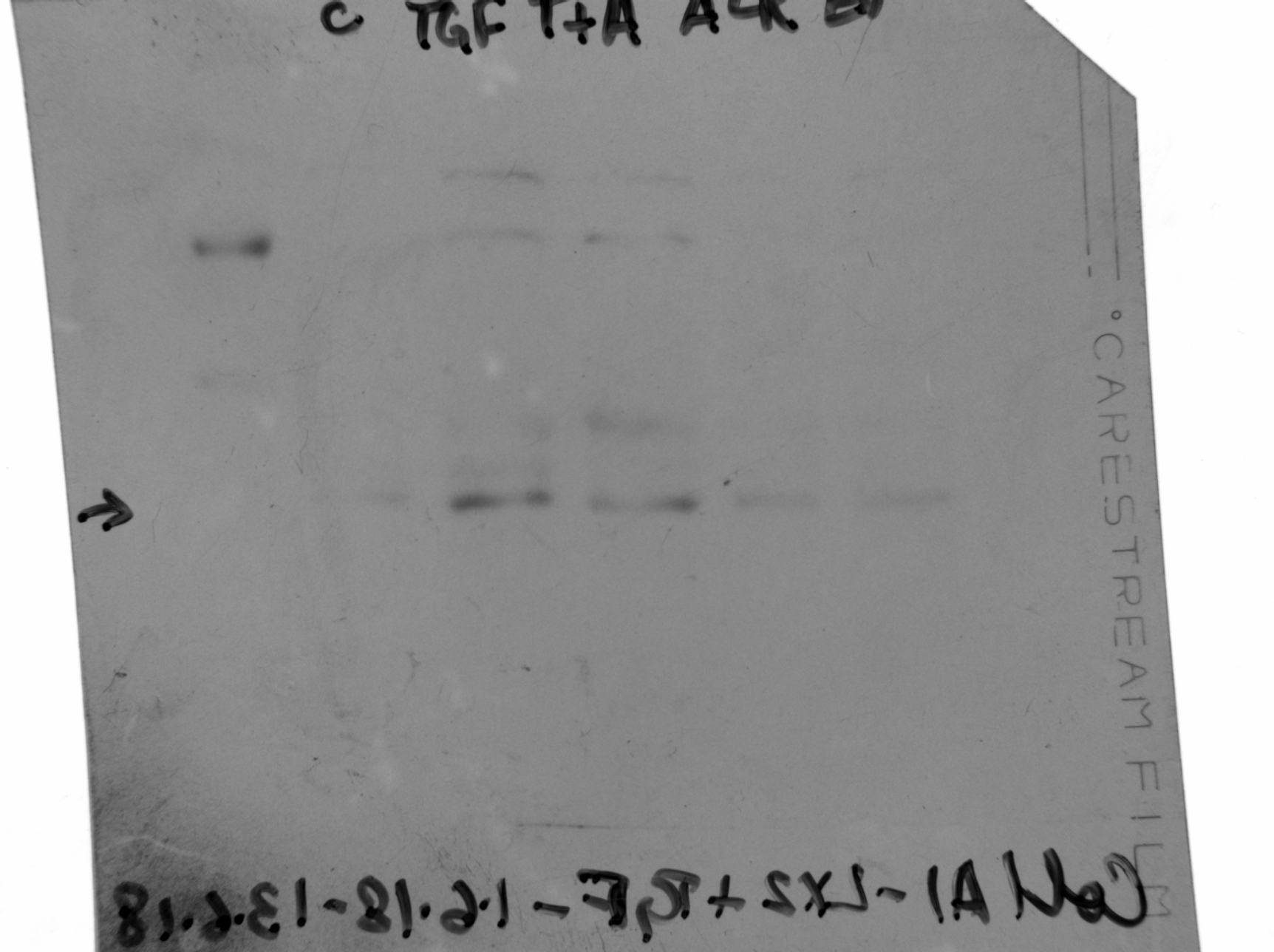

Supplement: S1 Dataset — (ZIP) [file pone.0214534.s001.zip › S1 dataset/Fig.2c_col1a1.tif]

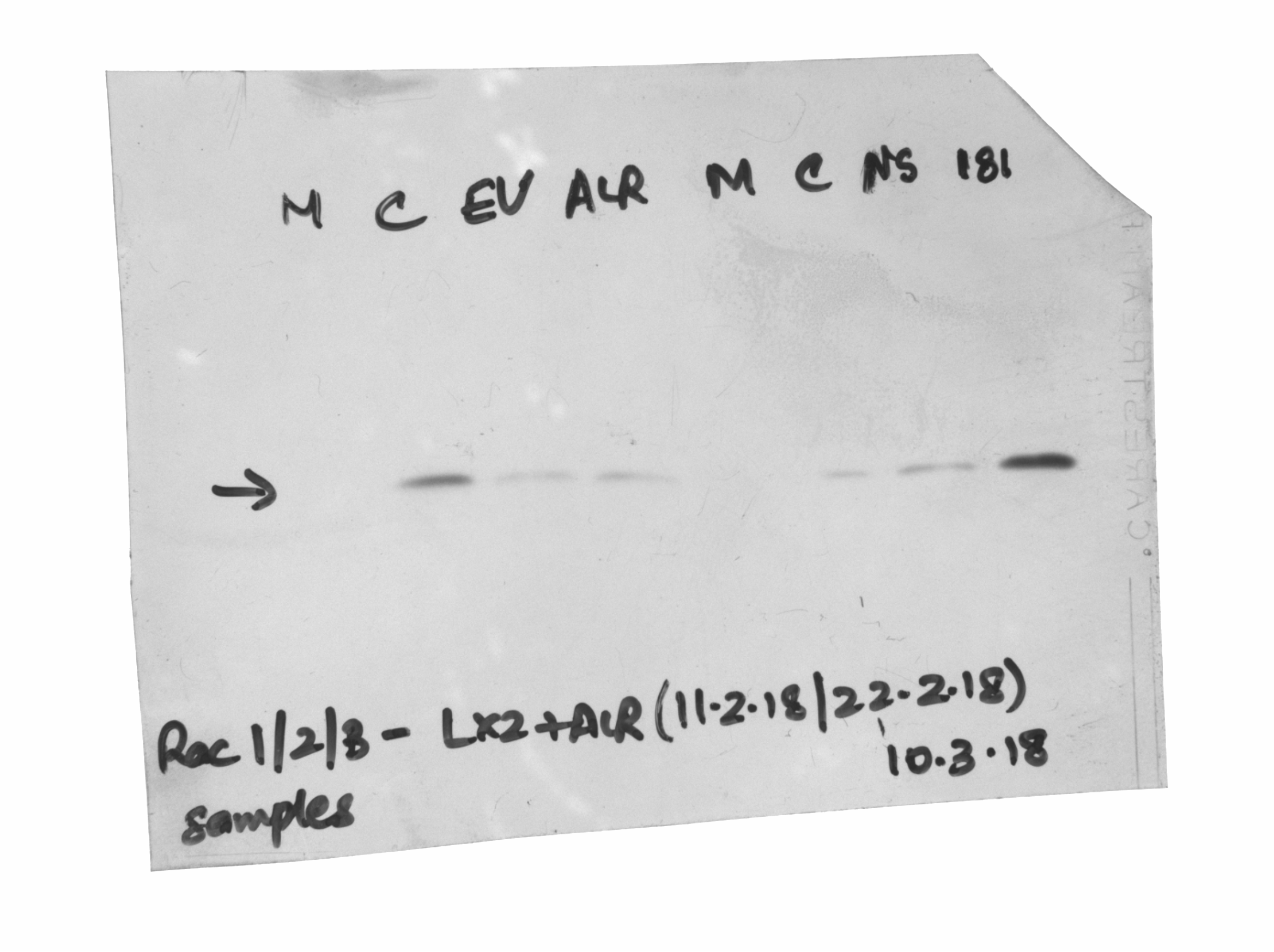

Supplement: S1 Dataset — (ZIP) [file pone.0214534.s001.zip › S1 dataset/Fig.3a_rac 1.tif]

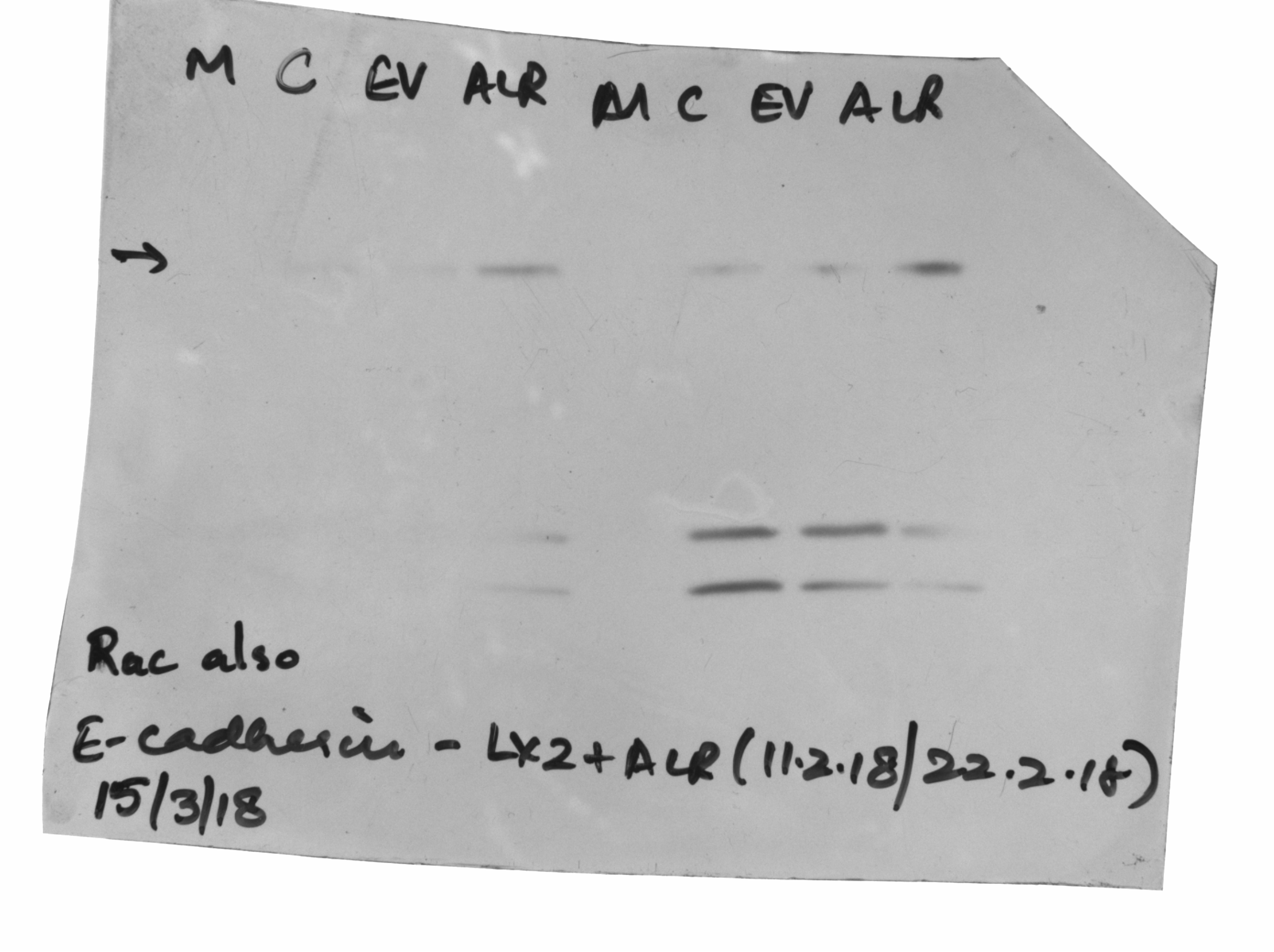

Supplement: S1 Dataset — (ZIP) [file pone.0214534.s001.zip › S1 dataset/Fig.5a n 5c_e-cadherin+rac 1.tif]
